# Association of Menstrual Extension and Surgery Effectiveness with Ultrasound Parameters of Cesarean Section Scar Diverticulum in Patients Undergoing Transvaginal Uterine Diverticulum Repair

**DOI:** 10.1155/2019/7415891

**Published:** 2019-12-19

**Authors:** Qing Yang, Min Ren, Xiaoli Lv, Fenghua Chen

**Affiliations:** Shanghai First Maternity and Infant Hospital, Tongji University School of Medicine, Shanghai, China

## Abstract

The association of residual myometrium thickness (RMT) and scar defect depth (*D*) with menstrual abnormalities and the effectiveness of vaginal repair remain to be determined in patients with cesarean section scar diverticulum (CSD). To assess the value of ultrasound to predict vaginal repair effectiveness. This was a retrospective study of patients with CSD treated with vaginal repair between 01/2014 and 02/2016 at Shanghai First Maternity and Infant Hospital (Tongji University). Transvaginal ultrasound was performed before and 3 months after surgical repair. RMT, *D*, scar defect length (*L*), and scar defect width (*W*) were measured. Width (*W*), *D*, and *L* increased along the duration of menstrual period (*P* < 0.05). When the menstrual extension time was ≥15 days, RMT/*D* and RMT/(RMT + *D*) were smaller than in patients with period <15 days (*P* < 0.05). *L* was the most positively correlated ultrasonic parameter with menstrual prolongation (*r* = 0.492). RMT/*D* and RMT/(RMT + *D*) were negatively correlated with prolonged menstruation (*r* = ‐0.304 and -0.305, respectively). RMT/*D* and RMT/(RMT + *D*) were associated with the disappearance of CSD after vaginal repair (*P* < 0.05). The cutoff value of RMT/(RMT + *D*) was 0.496, with sensitivity of 53.0% and specificity of 61.4%. *L* of CSD is closely correlated with menstrual extension but has no relationship with the effectiveness of surgery. RMT/(RMT + *D*) is correlated with menstrual extension time ≥15 days and the effectiveness of vaginal repair.

## 1. Introduction

In 1985, the World Health Organization (WHO) proposed that the ideal cesarean section (CS) rate should be 10%-15%, but over the past 30 years, the CS rate has gradually increased worldwide [1]. Despite the fact that CS is often necessary to save the neonate and the mother, many CS are performed for nonmedical reasons [2]. This is of concern because CS can result in many complications such as uterine rupture during the following pregnancy, chronic pelvic pain, and CS scar diverticulum (CSD) [3, 4]. CSD can cause prolonged menstrual period, menorrhagia, dysmenorrhea, and infertility [5, 6].

It is globally accepted that the first choice for assessing the CS scar is transvaginal ultrasonography [7, 8]. Vaginal repair of CSD is a common surgical method to restore the uterine anatomical morphology. Wang et al. [6] observed that abnormal symptoms of CSD were related to the width (*W*) of the scar defect but were unrelated with the residual myometrium thickness (RMT) and scar defect depth (*D*), and later agreed that the equation RMT/(RMT + *D*) offers additional information on the correlation between defect size and clinical symptoms [9].

Some studies have been reported the relationship between CSD size and menstrual abnormalities [5, 10–13], but beside the study by Wang et al. [6], little information is available on the relationship between menstrual abnormalities and RMT and *D*, as summarized by the equation RMT/(RMT + *D*). The exact association of RMT/(RMT + *D*) with menstrual abnormalities and the effectiveness of vaginal repair remain to be determined.

Therefore, the purpose of the present study was to examine the relationship between ultrasound parameters of CSD and menstrual abnormalities and to assess whether the ultrasound parameters could be used to predict the effectiveness of vaginal repair.

## 2. Methods

### 2.1. Study Design and Patients

This was a retrospective study of patients with CSD treated with vaginal repair between January 2014 and February 2016 at the Shanghai First Maternity and Infant Hospital affiliated to Tongji University. This study was approved by the Ethics Committee of the Shanghai First Maternity and Infant Hospital affiliated to Tongji University. The need for individual consent was waived by the committee.

The inclusion criteria were the following: (1) underwent at least one CS delivery, (2) CSD diagnosis by transvaginal ultrasound, and (3) underwent vaginal repair. The exclusion criteria were the following: (1) patients with any history of irregular periods, (2) use of intrauterine device, (3) use of hormonal contraceptives, (4) coagulation disorders, or (5) with any other uterine disease.

### 2.2. Ultrasound Examinations

Ultrasound examinations were performed using a Philips HD15 (US) system (Philips, Best, Netherlands) or a GE Voluson E8 system (GE Healthcare, Zipf, Austria). Both ultrasound devices were equipped with a 4-9 MHz transvaginal probe. The patient was in the lithotomy position after emptying the bladder. The transvaginal probe was used to gently touch the cervix to measure the uterine size and endometrial thickness. The multisection dynamic scan was performed to observe the location and morphology of the uterus, myometrium, endometrial echo, morphological changes, whether there were abnormal masses and effusions in the uterine cavity, and the presence or absence of abnormal echo in parametrial tissues. The characteristics of the CS scar at the lower segment of the uterine anterior wall (including the presence or absence of CSD, morphology of CSD, internal echo, relation with uterine cavity, and continuity of the muscular and serosal layers) were recorded. Auxiliary color Doppler ultrasonography was also performed.

**Figure 1:**
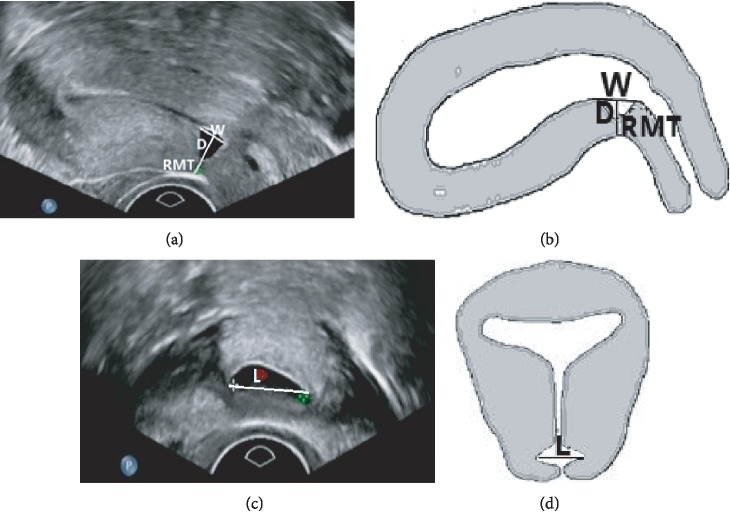
Ultrasonographic imaging of CSD before transvaginal repair surgery. (a) Ultrasound imaging of a cesarean scar diverticulum (CSD) on the sagittal plane. (b) Schematic diagram of the CSD on the sagittal plane. (c) Ultrasound imaging of a CSD on the transverse plane. (d) Schematic diagram of the CSD on the transverse plane. *W*: width of the scar niche on the sagittal plane; *D*: depth of the scar niche on the sagittal plane; RMT: residual myometrial thickness on the sagittal plane; *L*: length of the scar niche on the transverse plane.

Transvaginal ultrasound was routinely performed twice: before surgery and 3 months after surgical repair. For standardization purposes, the terms proposed by Naji et al. [8] were used in the present study. All ultrasound reports and images were reviewed to ensure the consistency of the definitions and values. The standardized approaches for imaging and measuring CSD were used, as described by Pomorski et al. [14] and Naji et al. [8]. Three parameters were measured on the sagittal plane: width of the hypoechoic scar niche (*W*), depth of the hypoechoic scar niche (*D*), and RMT (Figures 1(a) and 1(b)). The length of the hypoechoic scar niche (*L*) was measured on the transverse plane (Figures 1(c) and 1(d)). In addition, RMT/*D* and RMT/(RMT + *D*) were calculated.

### 2.3. Vaginal Repair

Transvaginal repair surgery was performed according to the previous studies [11, 15, 16]. All operations were performed by a chief professor with extensive experience in gynecological surgery. The patients underwent transvaginal diverticular repair and received transvaginal ultrasound examination 3 months after surgery to confirm the success of surgery. According to whether the anatomical structure of the cesarean section was restored, the patients were classified into two groups: the cured group (no diverticulum) and the unhealed group (with residual diverticulum).

### 2.4. Statistical Analysis

Statistical analysis was performed using SPSS 22.0 for Windows (IBM, Armonk, NY, USA). Continuous data are presented as means ± standard deviation or medians (range) and were analyzed using one-way analysis of variance (ANOVA) test or Kruskal-Wallis *H* test (intragroup comparisons) and the Student *t*-test or Mann–Whitney *U* test (intergroup comparisons), based on the results of the Kolmogorov-Smirnov test for normal distribution. Categorical data were presented as number and percentages and were analyzed using the chi-square test or the Fisher exact test, as appropriate. Correlations were analyzed with Spearman's rank correlation coefficient. The receiver operating characteristic (ROC) curve analysis was performed to determine the cutoff values. *P* values < 0.05 were considered statistically significant.

## 3. Results

### 3.1. Patient Characteristics

The study included 241 women with a mean age of 32.9 ± 3.7 (from 24 to 42) years. Among those, 177 (73.4%) women had undergone one CS, 61 (25.3%) had undergone two CS, and 3 (1.24) had undergone three CS. There were 230 (95.4%) patients with prolonged menstrual period, 8 (3.3%) with normal menstruation, 2 (0.8%) with midmenstrual menorrhagia, 1 (0.4%) with shortened menstrual cycle, and 3 (1.24%) with other conditions (2 had midmenstrual hemorrhage, and one had shortened period) (Table 1).

### 3.2. Ultrasound

**Figure 2:**
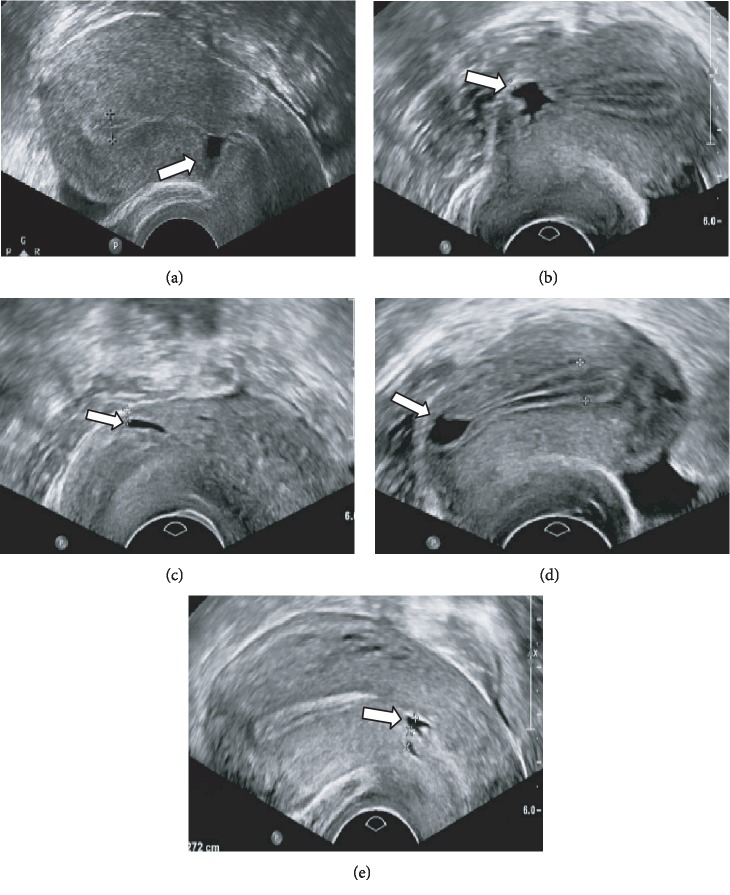
Shapes of the cesarean section diverticulum. (a) Triangle shape. (b) Wedge shape. (c) Quasicircular shape. (d) Droplet shape. (e) Irregular shape.

The shape of the diverticulum was roughly divided into five types: triangle (*n* = 102, 42.3%), wedge shape (*n* = 67, 27.8%), quasi-circular (*n* = 38, 15.8%), droplet (*n* = 19, 7.9%), and irregular shape (*n* = 15, 6.2%). The uterine position was categorized as retroflexion (*n* = 141, 58.5%), anteflexion (*n* = 91, 37.8%), and neutral position (*n* = 9, 3.7%) (Figure 2).

### 3.3. Factors Associated with Prolonged Menstruation

The patients with menstrual prolongation were divided into three subgroups: menstrual time 8-10 days, 11-14 days, and ≥15 days. The associations between prolonged menstrual bleeding, age, number of CS, and ultrasound parameters of CSD are presented in Table 2. The number of CS was higher in women with menstruations ≥15 days. *D*, *L*, and *W* progressively increased with the length of the menstrual period. RMT/*D* and RMT/(RMT + *D*) were smaller in patients with menstrual period ≥15 days.

### 3.4. Correlation between Ultrasound Parameters of CSD and Prolonged Menstruation

*L* was positively correlated with menstrual extension (*r* = 0.492). RMT/*D* and RMT/(RMT + *D*) were negatively correlated with prolonged menstrual period (*r* = ‐0.304) (Table 3).

### 3.5. Ultrasound Parameters of CSD and Surgical Repair Outcomes

**Figure 3:**
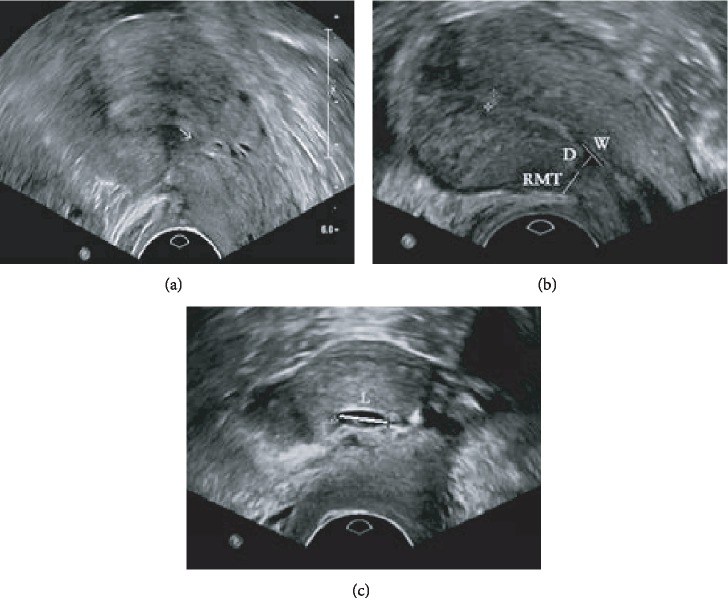
Ultrasonographic imaging of cesarean scar diverticulum (CSD) after transvaginal repair surgery. (a) Ultrasound imaging of CSD disappearance on the sagittal plane. No defect is seen in the scar shown by the arrow. (b) Ultrasound imaging showing CSD remained on the sagittal plane. (c) Ultrasound imaging showing CSD remained on the transverse plane. *W*: width of the scar niche on the sagittal plane; *D*: depth of the scar niche on the sagittal plane; RMT: residual myometrial thickness on the sagittal plane; *L*: length of the scar niche on the transverse plane.

After surgical repair, 124 patients' CSD disappeared while 117 patients' CSD remained. Ultrasonographic imaging of CSD after transvaginal repair surgery is shown in Figure 3.

Analysis of factors related to CSD existence or disappearance after surgery is performed in Table 4. There were significant differences in RMT/*D* (*P* = 0.048) and RMT/(RMT + D) (*P* = 0.048) before surgery between the absence or presence of CSD after surgery, but no differences in the number of CS, uterine position, *W*, *D*, *L*, and RMT (*P* < 0.05).

ROC curve was used to calculate the cutoff values of RMT/(RMT + *D*) for the absence or presence of CSD. RMT/(RMT + *D*) >0.496 indicated that the likelihood of CSD disappearance after VR was greater than 50%, with sensitivity of 53.0% and specificity of 61.4%.

## 4. Discussion

The association of RMT and *D* with menstrual abnormalities and the effectiveness of vaginal repair remain to be determined in patients with CSD. Therefore, the aim of the present study was to examine the relationship between ultrasound of CSD and menstrual abnormalities and to assess the value of ultrasound to predict vaginal repair effectiveness. The results suggest that the *L* of CSD is correlated with menstrual extension but has no relationship with CSD disappearance or existence after vaginal repair. RMT/(RMT + *D*) is correlated with menstrual extension time ≥15 days and the effectiveness of vaginal repair.

CS is an important means of dealing with high-risk pregnancies and solving difficult births [1]. Nevertheless, there are potential long-term risks for subsequent pregnancy such as scar pregnancy, placenta previa, and uterine rupture [3, 4]. In recent years, the incidence of CS has gradually increased [1]. A survey of 39 hospitals in Mainland China showed that the incidence of CS is 54.5%, among which the CS rate without indication was 24.6% [17]. With the increase of cesarean section rate, the incidence of CSD also increases gradually, which is reported to be as high as 62.5% among women with at least one CS [17].

The factors leading to poor CS scar healing are unclear. From nine studies, Melo-Cerda et al. classified all risk factors into four main categories: closure technique, development of the lower uterine segment or location of the incision, wound healing, and miscellaneous [18]. The risk of CS scar increases in women with a retroflexed uterus and in those who had undergone multiple CS [19]. Wang et al. [6] found that multiple CS and retroflexed uterus were risk factors for larger CSD. In the study by Osser et al. [20], the frequency of large scar defects increased with the number of CS [20]. Some authors suggested that CSD development was significantly associated with premature rupture of membrane and short operation time [21]. In the present study, 141 cases of retroflexed uterus (58.5%) were reported.

Currently, there is no uniform diagnostic standard for CSD. For patients with clinical symptoms, it is necessary to exclude endocrine factors and examine the uterus carefully to make a diagnosis. At present, the examinations of CSD include transvaginal ultrasound, magnetic resonance imaging, hysteroscopy, hysterosalpingography, and saline contrast sonohysterography. Transvaginal ultrasound can be used to measure and describe the morphology of CS scars when present [7, 8]. Transvaginal ultrasound is a first-line clinical tool for the diagnosis of abnormal uterine bleeding [22] and is the first choice for the noninvasive examination of CSD [5, 23]. In the present study, the CS scars in transvaginal ultrasound were classified into triangle (42.3%), wedge (27.8%), quasicircular, droplet, and irregular shapes. Park et al. [21] reported that the triangle and quasicircular shapes were the most common, supporting the present study.

Using vaginal ultrasonography, Wang et al. [6] observed that the *W* of CSD was associated with postmenstrual spotting, but was unrelated to the RMT, supporting the present study. We also found that the *W*, *D*, and *L* of CSD were related to menstrual extension. In other words, the wider, the deeper, and the longer the CSD is, the longer the length of menstrual prolongation is. Among those, *L* was the most closely related to the duration of menstrual prolongation. Interestingly, we also found that although RMT had no relationship with prolonged menstruation, RMT/*D* and RMT/(RMT + *D*) were associated with prolonged menstruation ≥15 days.

The methods for the surgical treatment of CSD include transvaginal diverticulum repair, hysteroscopy, and laparoscopy. Transvaginal repair is an effective minimally invasive surgical treatment [10, 15, 24–26]. Among the studies about the factors associated with surgical success, only one suggested that multiple CS and CSD volume >600 mm^3^ measured by magnetic resonance imaging were risk factors for surgical failure [27]. Pomorski et al. [14] reported that *D*/RMT was useful to predict the occurrence of scar dehiscence in the next pregnancy. In the present study, RMT/*D* could also predict the surgical outcome: the larger RMT and the smaller *D* are, the greater the possibility of surgical cure is. We were surprised to find that none of the assessed CSD parameters individually was useful for predicting outcome of surgery. One possible explanation is that RMT and *D* values together represent the complete muscular thickness of the scar site of a CS. RMT/(RMT + *D*) can be calculated to provide an indication of the percentage of RMT at the exact location of the CS scar.

This study has limitations. The sample size was relatively small and from a single center, and all were operated by the same surgeon. The clinical experience and suturing techniques of surgeons during transvaginal repair are central to surgical success [28], and they cannot be measured using standard quantitative indicators. Furthermore, the vascular perfusion oxygenation in the scar tissue [29] may need to be taken into account in future studies on the effectiveness of CSD surgery.

## 5. Conclusions

The results of the present study suggest that RMT/(RMT + *D*) is an important parameter in ultrasound assessment of CSD. RMT/(RMT + *D*) is related not only to prolonged menstruation ≥15 days but also to the outcome of transvaginal CSD repair. Those findings may help in counselling for clinical symptoms and surgical decision of patients with CSD. Future studies on surgical may need to take these into account.

## Figures and Tables

**Table 1:** Characteristics of the patients.

Parameters	*n*	Value
Age (years)	241	32.9 ± 3.7
Number of CS		
1	177	
2	61	
3	3	
Age of CS (years)		
1	241	26.4 ± 3.8
2	61	28.7 ± 3.7
3	3	27.5 ± 2.1
Menstrual before first CS (days)	241	6.2 ± 1.0
Menstrual changes		
Prolonged bleeding	230	14.4 ± 3.3
Normal	8	
Other	3	
Uterine position		
Retroflexion	141	
Anteflexion	91	
Neutral position	9	
Last CS to surgery interval (years)	241	5.0 ± 2.8

CS: cesarean section.

**Table 2:** Relationship between prolonged menstrual bleeding, age, number of CS, and ultrasound parameters of CSD.

	8-10 days	11-14 days	≥15 days	*P*
	*N* = 56	*N* = 78	*N* = 96	
Age (years) (mean ± SD)	32.3 ± 3.4	33.1 ± 3.9	33.2 ± 3.9	0.303
Number of CS				0.008
1	43	65	59	
2	13	13	34	
3	0	0	3	
Uterine position				0.108
Retroflexion	35	50	47	
Anteflexion	21	24	45	
Neutral position	0	4	4	
*W*	5 (4-7)	7 (6-9)^#^	8 (6-11)^#,^^∗^	<0.001
*D*	5 (4-7)	7 (5-8)^#^	8.5 (6-10)^#,^^∗^	<0.001
*L*	9.3 ± 3.9	12.5 ± 4.1^#^	16.6 ± 4.7^#,^^∗^	<0.001
RMT	2.4 (2-3.42)	2.4 (2-3)	2.3 (1.7-2.92)	0.177
RMT/*D*	0.50 (0.33-0.75)	0.40 (0.27-0.57)	0.28 (0.19-0.43)^#^^∗^	<0.001
RMT/(RMT + *D*)	0.33 (0.25-0.43)	0.29 (0.21-0.36)	0.22 (0.16-0.30)^#,^^∗^	<0.001

CS: cesarean section; CSD: cesarean section scar diverticulum; *W*: width of the niche; *D*: depth of the niche; *L*: length of the niche; RMT: residual myometrial thickness. ^#^*P* < 0.05 vs. 8-10 days and ^∗^*P* < 0.05 vs. 11-14 days.

**Table 3:** Correlation coefficients of variables related to prolonged menstruation.

*N* = 230	Correlation coefficient	*P*
Number of CS	0.193	0.003
*W*	0.323	<0.001
*D*	0.327	<0.001
*L*	0.492	<0.001
RMT/*D*	-0.305	<0.001
RMT/(RMT + *D*)	-0.304	<0.001

CS: cesarean section; *W*: width of the niche; *D*: depth of the niche; *L*: length of the niche; RMT: residual myometrial thickness.

**Table 4:** Characteristics of CSD disappearance and existence after vaginal repair.

Variables	CSD disappearance after VR	CSD existence after VR	*P*
	*N* = 124	*N* = 117	
Age (years) (mean ± SD)	32.8 ± 3.8	33.1 ± 3.7	0.495
Number of CS			0.909
1	92	86	
2	31	29	
3	1	2	
Uterine position			0.29
Retroflexion	73	68	
Anteflexion	49	42	
Neutral position	2	7	
*W*	7 (5-9)	7 (5-11)	0.147
*D*	7 (5-9)	7 (5.5-9)	0.237
*L*	12.9 ± 5.2	13.8 ± 5.3	0.196
RMT	2.5 (2.0-3.0)	2.2 (1.8-3.0)	0.088
RMT/*D*	0.40 (0.26-0.60)	0.31 (0.23-0.50)	0.048
RMT/(RMT + *D*)	0.29 (0.20-0.38)	0.24 (0.19-0.33)	0.048

CS: cesarean section; CSD: cesarean section scar diverticulum; W: width of the niche; D: depth of the niche; L: length of the niche; RMT: residual myometrial thickness.

## Data Availability

No data were used to support this study.
